# A Metric on Phylogenetic Tree Shapes

**DOI:** 10.1093/sysbio/syx046

**Published:** 2017-05-02

**Authors:** C. Colijn, G. Plazzotta

**Affiliations:** *Department of Mathematics, Imperial College, 180 Queen’s Gate, London SW7 2AZ, UK*

**Keywords:** tree metric, phylodynamics, tree shapes

## Abstract

The shapes of evolutionary trees are influenced by the nature of the evolutionary process but comparisons of trees from different processes are hindered by the challenge of completely describing tree shape. We present a full characterization of the shapes of rooted branching trees in a form that lends itself to natural tree comparisons. We use this characterization to define a metric, in the sense of a true distance function, on tree shapes. The metric distinguishes trees from random models known to produce different tree shapes. It separates trees derived from tropical versus USA influenza A sequences, which reflect the differing epidemiology of tropical and seasonal flu. We describe several metrics based on the same core characterization, and illustrate how to extend the metric to incorporate trees’ branch lengths or other features such as overall imbalance. Our approach allows us to construct addition and multiplication on trees, and to create a convex metric on tree shapes which formally allows computation of average tree shapes.

The availability and declining cost of DNA sequencing mean that data on the diversity, variation and evolution of organisms is more widely available than ever before. Increasingly, thousands of organisms are being sequenced at the whole-genome scale (Chewapreecha et al. 2014; Bedford et al. 2015; Anopheles gambiae 1000 Genomes 2016). This has had particular impact on the study of pathogens, whose evolution occurs rapidly enough to be observed over relatively short periods. As the numbers of sequences gathered annually grow to the tens of thousands in many organisms, comparing this year’s evolutionary and diversity patterns to previous years’, and comparing one location to another, has become increasingly challenging. Despite the fact that evolution does not always occur in a tree-like way due to the horizontal movements of genes, phylogenetic trees remain a central tool with which we interpret these data.

The shapes of phylogenetic trees are of long-standing interest in both mathematics and evolution (Slowinski 1990; Guyer and Slowinski 1993; Kirkpatrick and Slatkin 1993; Mooers and Heard 1997; Stam 2002; Blum and François 2006; Purvis et al. 2011; Wu and Choi 2015). A tree’s shape refers to the tree’s connectivity structure, without reference to the lengths of its branches. A key early observation was that trees reconstructed from evolutionary data are more asymmetric than simple models predict (Aldous 1996). This spurred an interest in ways to measure tree asymmetry (Kirkpatrick and Slatkin 1993; Fusco and Cronk 1995; Aldous 2001; Stich and Manrubia 2009; Pompei et al. 2012), in the power of asymmetry measures to distinguish between random models (Kirkpatrick and Slatkin 1993; Agapow and Purvis 2002; Matsen 2006), and in constructing generative models of evolution that produce imbalanced trees (Aldous 2001; Blum and François 2006; Manceau et al. 2015). Tree shapes carry information about the underlying evolutionary processes, and distributions of tree shapes under simple null models can be used to test hypotheses about evolution (Mooers and Heard 1997; Blum and François 2006; Blum et al. 2006; Purvis et al. 2011; Wu and Choi 2015). Recent work also relates fitness, selection and a variety of ecological processes to tree shape (Gascuel 2000; Hein et al. 2004; Maia et al. 2004; Wakeley and Wakeley 2009; Dayarian and Shraiman 2014; Manceau et al. 2015). An additional motivation for studying the shapes of phylogenetic trees is that reconstructing branch lengths is challenging, particularly deep in a tree. There may be weak support for a molecular clock, and coalescent inference procedures may produce trees with consistent shape but differing root heights.

Tree shape is well established as carrying important information about macroevolutionary processes, but also carries information about evolution in the short term. In the context of pathogens, diversity patterns represent a combination of neutral variation that has not yet become fixed, variation that is under selection, complex demographic processes (host behavior and contact patterns), and an array of ecological interactions. The extent to which tree shapes are informative of these processes is not well understood, though there have been studies on the frequency of cherries and tree imbalance (Volz et al. 2013; Lambert and Stadler 2013; Plazzotta et al. 2016) and simulation studies aiming to explore the question (Leventhal et al. 2012; Robinson et al. 2012; Colijn and Gardy 2014; Plazzotta and Colijn 2016).

A key limitation in relating tree shapes to evolution and ecology has been the limited tools with which trees can be quantified and compared. Comparing tree shapes from different models of evolution or from different data sets requires comparing *unlabeled* trees, whereas most tree comparison methods (e.g., (Robinson and Foulds 1981), Billera-Holmes-Vogtmann (Billera et al. 2001) and newer metrics (Kendall and Colijn 2016)) compare trees with one particular set of organisms at the tips (one set of taxa, with the labels in each tree). These metrics can be used as a basis for metrics on unlabeled shapes, for example by setting the distance between shapes $T_1$ and $T_2$ to be $d(T_1,T_2)=min(d_{rf}(\hat{T}_1,\hat{T}_2))$, where $\hat{T}_i$ has shape $T_i$ and the Robinson Foulds distance is computed by labeling $\hat{T}_1$ and $\hat{T}_2$ with the same set of labels. However, this requires computing the distance using every distinct arrangement of tip labels on one of the trees. Similarly defined metrics on trees with multisets for their labels have been described (Huber et al. 2011), but their computation is difficult and metrics may not be applicable if the trees have different numbers of tips. Consequently, the tools at our disposal to describe and compare tree shapes from *different* sets of tips are limited, and have focused on scalar measures of overall asymmetry (Sackin 1972; Slowinski 1990; Guyer and Slowinski 1991; Colless 1995; Fusco and Cronk 1995; Matsen 2006; Stich and Manrubia 2009; Pompei et al. 2012) and on the frequencies of small subtree shapes such as cherries (Steel and McKenzie 2000; Volz et al. 2013; Plazzotta and Colijn 2016) and r-pronged nodes (Rosenberg 2006). Recently, kernel (Poon et al. 2013) and spectral (Lewitus and Morlon 2015) approaches also have been used.

Here we give a simple characterization of all possible shapes for a rooted tree and use this to define metrics (in the sense of true distance functions) on tree shapes. The scheme provides an efficient way to count the frequencies of sub-trees in large trees, and hence can be used to compare empirical distributions of sub-tree shapes. It is not limited to binary trees and can be formulated for any maximum size multifurcation, as well as for trees with internal nodes with only one descendant (sampled ancestors). As an illustrative example, we apply a metric derived from our scheme to simulated and data-derived trees. Our scheme and our metric separate trees from random tree models that are known to produce trees with different shape. We use the approach to compare trees from tropical versus USA human influenza A (H3N2). We extend the metric to incorporate statistics on the lengths of branches or other tree features, and we use a map from tree shapes to the rational numbers to define a convex metric on tree shapes.

## Materials and Methods

### Definitions

A *tree shape* is a tree (a graph with no cycles), without the additional information of tip labels and branch lengths. We consider rooted trees, in which there is one node specified to be the root. Tips, or leaves, are those nodes with degree 1. A *rooted tree shape* is a tree shape with a vertex designated to be the root. We use "tree shape," as we assume rootedness throughout. Typically, edges are implicitly understood to be directed away from the root. A node’s *children* are the node’s neighbors along edges away from the root; each node is the *parent* of its children. In a binary tree shape, the root has two children and is the only node without a parent. A *multifurcation*, or a *polytomy*, is a node with more than two children, and its *size* is its number of children ($>2$). Naturally, a rooted phylogeny defines a (rooted) tree shape if the tip labels and edge weights are discarded. Phylogenies typically do not contain internal nodes with fewer than two children (sampled ancestors), but we allow this possibility.

### Labeling Scheme

**Figure 1:**
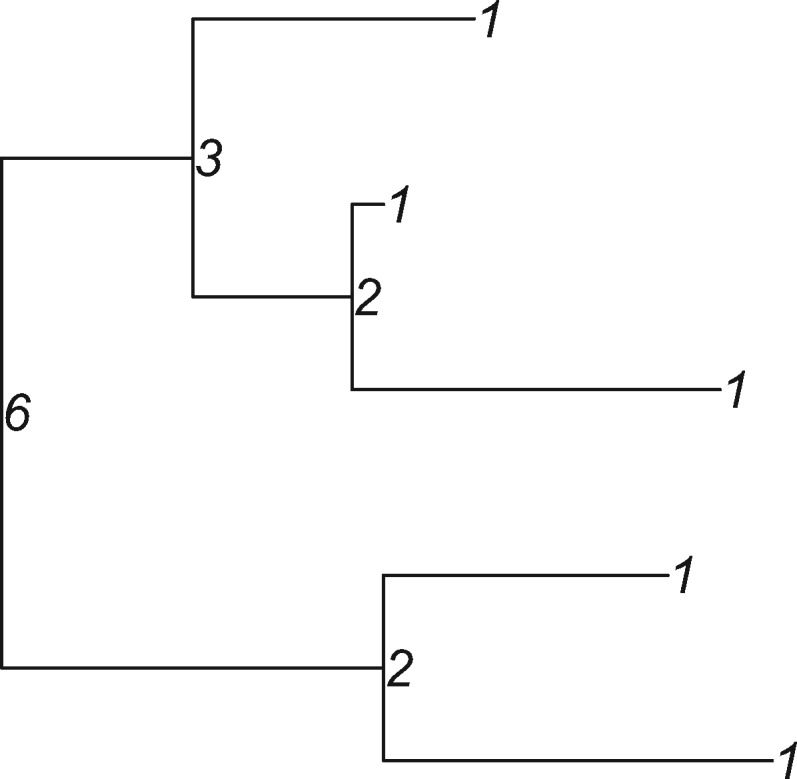
Example of the labels on a five-tip tree.

Our approach is to label any possible tree shape, traversing the tree from the tips to the root and assigning labels as we go. The simplest case is to assume a binary tree, in which all internal nodes have two children. We start by giving all tips the Label $1$ and proceed up the tree moving from the tips to the root. We use the labels of each node’s children to define that node’s label. So for every internal node, we list its childrens’ labels $(k,j)$, organizing them with lexicographic sorting (i.e., listing with the larger of $k$ and $j$ first and then in increasing order, very like alphabetical sorting). The lexicographically sorted list of all $(k,j)$ pairs is: $(1)$, $(1,1)$, $(2,1)$, $(2,2)$, $(3,1)$, $(3,2)$, $(3,3)$, $(4,1)$, $(4,2)$, $(4,3)$, $(4,4)$, $(5,1) ...$ We define the label of a tree shape whose root node has children $(K,J)$ to be the index at which $(K,J)$ appears in this list. Accordingly, a “cherry” (a node with two tip children) is labeled $2$ because its children are $(1,1)$, which is the second entry in the list. A node with a cherry child and a tip child (a $(2,1)$, or a pitchfork) has Label 3. A tree whose root has children labeled $4$ and $2$ (4,2) is the 9${th}$ item in the list and so has Label 9. As we traverse the tree from the tips to the root, we label each internal node using the labels of its children. While Labels 1, 2, and 3 coincidentally are trees with 1, 2, and 3 tips, this correspondence is soon lost because there are many trees with $n$ tips in general; there are two possible trees with 4 tips: a “double cherry” (2,2) with Label 4, and a (3,1), with Label 5. A small example is shown in Figure 1.

The binary tree shape $(k,j)$ (a tree whose root has a child with label $k$ and one with label $j$) has label

(1) $$\phi_{2}(k,j)=\frac{1}{2}k(k-1)+j+1$$

because $(k,j)$ is the $\tfrac{1}{2} k (k-1)+j+1$’th entry in the lexicographically sorted list. To see this, note that for some fixed $k$, there are $k$ pairs of the form $(k,j)$ (because $j$ ranges from 1 to $k$). This means that the pair $(n,1)$ has label $(1+2+...+n-1)+1+1= \tfrac{1}{2} n(n-1)+1 + 1$, because all labels starting with $1, 2, 3, ... k, .. n-1$ occur before this first pair beginning with $n$. The extra 1 accounts for starting the scheme with $(1,1)$ whose label is 2. So $(k,j)$ has labeled $\tfrac{1}{2} k(k-1)+j +1$ as above. We continue until the root of the tree has a label. The subscript $2$ in Equation 1 specifies that each node has a maximum of 2 children; the scheme can be extended but has a different explicit form ($\phi_M$) if there are multifurcations up to size $M$ or internal nodes with a single child (in which case we require $j\geq 0$ rather than 1). We give details in the Supplementary Material available on Dryad at http://dx.doi.org/10.5061/dryad.3r8v1.

### Metrics on the Space of Rooted Unlabeled Shapes

This characterization leads to simple metrics on the space of tree shapes. The simplest is a comparison of the root labels: given two binary trees $T_a$ and $T_b$, whose root nodes are $R_a$ and $R_b$, and where the label of a node $x$ is $L(x)$, we can write

(2) $$d_{0}(T_{a},T_{b})=|L(R_{a})-L(R_{b})|.$$

In other words, the absolute difference between the root nodes’ labels is a metric, with tree 1 a distance of 1 from tree 2 and so on. Clearly $d_0$ is symmetric and non-negative. The tree isomorphism algorithm and the above labeling clearly show that $d_0=0 \Leftrightarrow T_a = T_b$ and the absolute value obeys the triangle inequality. However, $d_0$ is not a very useful metric, in the sense that a large change in root label can result from a relatively “small” change in the tree shape (such as the addition of a tip).

While each tree is defined by the label of its root, it is also defined (redundantly) by the labels of all its nodes. If the tree has $n$ tips, the list of its labels contains $n$ 1s, typically multiple 2s (cherries) and so on. Let $L_a$ denote the list of labels for a tree $T_a$: $L_a = \{ 1, 1, 1, ..., 2, 2, ..., \phi_2(R_a)\}$. The label lists are multisets because labels can occur multiple times. Define the distance $d_1$ between $T_a$ and $T_b$ to be the number of elements in the symmetric set difference between the label lists of two trees:

(3) $$d_{1}(T_{a},T_{b})=|L_{a}\Delta L_{b}|.$$

The symmetric set difference $ A \Delta B = ( A \cup B ) \backslash (A \cap B)$ is the union of $A$ and $B$ without their intersection. Intuitively, this is the number of labels not included in the intersection of the trees’ label lists. If $A$ and $B$ are multisets with $A$ containing $k$ copies of element $x$ and $B$ containing $m$ copies of $x$, with $k>m$, we consider $A\cap B$ to contain $m$ copies of $x$ (these are common to both $A$ and $B$). $A \Delta B$ has the remaining $k-m$ copies. Each tree’s label list contains more 1s (tips) than any other label. Accordingly, this metric is most appropriate for trees of the same size, because if trees vary in size, the metric can be dominated by differences in the numbers of tips. For example, if $L_a = \{ 1,1,1,1,2,2 \}$ (four tips joined in two cherries) and $L_b = \{ 1,1,1,2,3\}$ (three tips, i.e., a pitchfork), then $L_a \Delta L_b = \{1,2,3\}$, because there is a 1 and a 2 in $L_a$ in excess of those in $L_b$, and a $3$ in $L_b$, that is, not matched in $L_a$. Like $d_0$, $d_1$ is a metric: positivity and symmetry are clear from the definition. The cardinality of the symmetric difference is 0 if and only if the two sets are the same, in which case the root label is the same and the tree shapes are the same. That the symmetric difference obeys the triangle inequality is readily seen from the property $A \Delta C \subset (A \Delta B ) \cup (B \Delta C)$.

Perhaps the most natural metric based on the labels, and the metric that we apply (and extend) through this work, compares the numbers of occurrences of each label in each tree. Let $v_a$ be a vector whose $k'$th element $v_a(k)$ is the number of times label $k$ occurs in the tree $T_a$; so $v_a(1)$ will be the number of tips, $v_a(2)$ the number of cherries, and so on. Define the metric $d_2$ as the Euclidean norm (square root of sum of squares) of the difference between $v_a$ and $v_b$:

(4) $$d_{2}(T_{a},T_{b})=||v_{a}-v_{b}||.$$

Positivity, symmetry and the triangle inequality are evident, and again $d_2$ can only be 0 if $T_a$ and $T_b$ have the same number of copies of all labels (including the root label), which is true if and only if $T_a$ and $T_b$ have the same shape. This has a similar flavor to the statistic used to compare trees to Yule trees in (Blum and François 2006), where the numbers of clades of a specific size were compared. We have used and extended metric $d_2$ in the analyses presented in the Results section.

Each of these metrics is computed in linear time. If $T_a, T_b$ have $n_a, n_b$ internal nodes, computing the distance requires $O(n_a+n_b)$ operations to define the labels, and $O(\text{max}(n_a,n_b))$ operations to compare the lists of labels. Different choices of weights increase computational time but not computational complexity; the variants we present are all linear in the (maximum) number of tips of the two trees.

### Simulations

We compared trees from different random processes and models. One of the most natural random processes modelling phylogenetic trees is the continuous-time homogeneous birth–death (BD) branching process, in which each individual gives rise to a child at a constant rate in time, and also risks removal (death) at a constant rate. With birth rate $\lambda$ and death rate $\mu$, the ratio $\lambda/\mu$ specifies the mean number of offspring of each individual in this process, and affects the shapes and branching times of the resulting branching trees. In the epidemiological setting, the link to branching times has been used to infer the basic reproduction number $R_0$ from sequence data (Stadler et al. 2012, 2014). We computed the distances between trees derived from constant-rate BD processes simulated in the package TreeSim in R (Stadler 2017). One challenge is that the number of tips in the BD process after fixed time is highly variable and depends on $\lambda/\mu$. We aimed to detect shape differences that were not dominated by differences in the number of tips. Accordingly, we conditioned the processes to have 1500 taxa and then pruned tips uniformly at random to leave approximately 1250 tips remaining.

There are several other random models for trees. The Yule model is a model of growing trees in which lineages divide but do not die; in terms of tree shape it is the same as the Kingman coalescent and the equal rates Markov models. In the ‘proportional to distinguishable arrangements’ (PDA) model, each unlabeled shape is sampled with probability proportional to the number of *labeled* trees on $n$ tips with that unlabeled shape (Rosen 1978; Mooers and Heard 1997). The “biased” model is a growing tree model in which a lineage with speciation rate $r$ has child lineages with speciation rates $pr$ and $(1-p)r$. The Aldous’ branching model that we use here is Aldous’ $\beta$-splitting model with $\beta=-1$ (Aldous 1996); in this model a $\beta$ distribution determines the (in general asymmetric) splitting densities upon branching. The Yule, PDA, biased and Aldous $\beta=-1$ models are available in the package apTreeshape in R (Bortolussi et al. 2006). We used $p=0.3$ for the biased model, and sampled trees with 500 tips.

### Data

We aligned data of HA protein sequences from human influenza A (H3N2) in different settings reflecting different epidemiology. Data were downloaded from NCBI on 22 January 2016. In all cases, we included only full-length HA sequences for which a collection date was available. The USA data set ($n=2168$) included USA sequences collected between March 2010 and September 2015. The tropical data ($n=1388$) included sequences from the tropics collected between January 2000 and October 2015. Accession numbers are included in the Supporting Information. Fasta files were aligned with mafft. Within each data set, we sampled 500 taxa uniformly at random (repeating 200 times) and inferred a phylogenetic tree with the program FastTree (Price et al. 2009). Where necessary we realigned the 500 sequences before tree inference. This resulted in 200 trees, each with 500 tips from the tropical and USA isolates.

Note that this approach is distinct from Bayesian inference of many trees on *one* set of tips, and from bootstrap trees on one set of tips. Either a posterior or bootstrap collection of trees from the same set of tips will share shape features because of the phylogenetic signal in the data. In contrast, we resample from the isolate collection each time and the trees we compare do not have the same set of labels.

### Implementation

We have used R throughout. An R package is available on github at https://github.com/carolinecolijn/treetop. The implementation assumes full binary trees and includes metrics $d_1$ and $d_2$ with the option of weighting, as well as a “tree lookup” function that returns the tree associated with an integer in labeling scheme $\phi_2$.

## Results

### Label-Based Description of Tree Shapes

**Figure 2:**
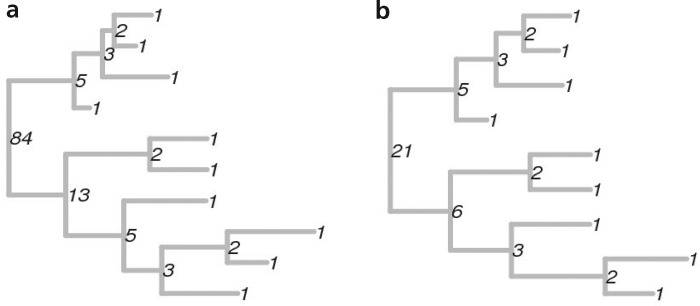
Illustration of the labels of the nodes of binary trees. Tips have the Label $1$. Labels of internal nodes are shown in black. The only difference between the trees in (a) and (b) is that in (b), the bottom-most tip from (a) has been removed. As a consequence, most of the labels are the same.

Figure 2 illustrates the labels at the nodes of two binary trees. The label of the root node uniquely defines the tree shape. Indeed, tree isomorphism algorithms use similar labeling (Hopcroft and Tarjan 1972; Lueker and Booth 1979; W 1979; Colbourn and Booth 1981; Sayward 1981). If $R_a$ and $R_b$ are the root nodes of binary trees $T_a$ and $T_b$, the tree shapes are the same if and only if $\phi_2(R_a)=\phi_2(R_b)$. The map between trees and labels is bijective: every positive integer corresponds to a unique tree shape and vice versa.

Metrics are an appealing way to compare sets of objects; defining a metric defines a *space* for the set of objects—in principle allowing navigation through the space, study of the space’s dimension and structure, and the certainty that two objects occupy the same location if and only if they are identical. The labeling scheme gives rise to several natural metrics on tree shapes, based on the intuition that tree shapes are similar when they share many subtrees with the same labels.

### Simulated Random Trees

**Figure 3:**
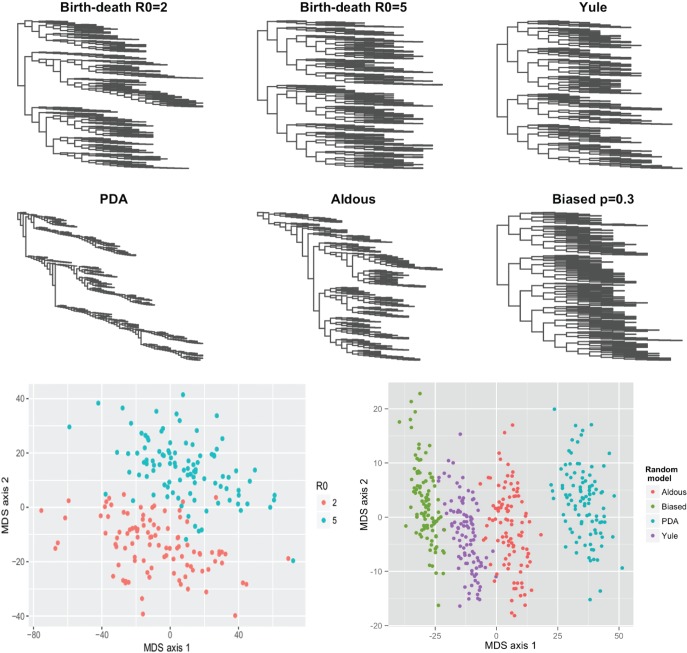
Top: Six sample trees, one from each of six different random processes. Bottom: Multi-dimensional scaling (MDS) plots showing that trees from each process are grouped together in the metric. Bottom left: trees from a BD model with different values of $R_0=\lambda/\mu$. Bottom right: trees derived from the Yule, PDA, Aldous and biased models, each with 500 tips.

There are several ways to sample random trees in ways known to produce trees of different shapes (in particular, different asymmetry). These include models capturing equal versus different speciation rates, continuous time BD processes with different rates and others (see Methods). We used the metric arising from our labeling scheme to compare these. Figure 3 shows a visualization of the tree-tree distances between trees from different random models. The metric groups trees from each process together and distinguishes between them well. Summary statistics such as tree imbalance also distinguish some of these groups well (particularly the PDA, Aldous, Yule and biased speciation model); indeed, we have elsewhere related the basic reproduction number to the number of cherries (Plazzotta and Colijn 2016), and because the cherry is a symmetric configuration, trees with a high frequency of cherries will be more symmetric than those with a low frequency of cherries.

### Tropical Versus Seasonal Influenza

**Figure 4:**
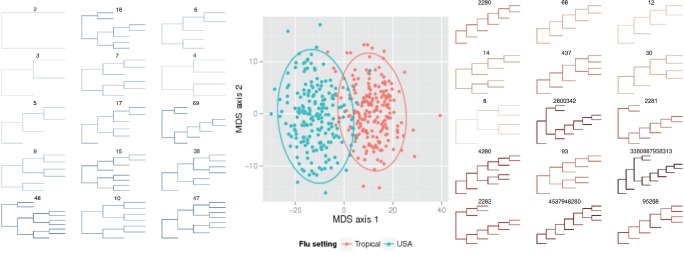
Comparisons between trees from H3N2 flu virus samples. Central panel: multi-dimensional scaling plot showing that the metric separates trees from the tropics (red) and from the USA (blue). Left and right panels: top-ranked sub-trees that distinguish the two groups, as determined by discriminant analysis of principal components (DAPC); labels correspond to the labeling scheme. Depth of color corresponds to Sackin imbalance (see Supplementary Material available on Dryad).

We also compared trees inferred from sequences of the HA protein in influenza A H3N2 sequences. Influenza A is highly seasonal outside the tropics (Russell et al. 2008), with the majority of cases occurring in winter. In contrast, there is little seasonal variation in transmission in the tropics. In addition, over long periods of time, influenza evolves in response to pressure from the human immune system, undergoing evolution particularly in the surface HA protein. This drives the ‘ladder-like’ shape of long-term influenza phylogenies (Koelle et al. 2010; Volz et al. 2013; Westgeest et al. 2012; Luksza and Lässig 2014), but would not typically be apparent in shorter-term data sets. With this motivation, we compared tropical samples to USA sample. Figure 4 shows that the tropical and USA flu trees are well separated by the metric. In addition, we used DAPC (Jombart et al. 2010) to determine which shapes separate the two groups. These shapes are those with high loadings on the first (and only substantial) principal component. We show them in Figure 4, listing their labels and coloring them according to Sackin imbalance. The two groups are different in imbalance, and the metric allows us to determine which sub-shapes occur with different frequencies to separate the groups. In the Supplementary Material available on Dryad, we compare the imbalance and numbers of cherries across the various groups of trees.

### Incorporating Tree Size, Branch Lengths, and Other Properties

Perhaps as it should be, the dominant difference between a tree with ten tips and one with one hundred tips is the size of the tree (and for this reason we have focused our application on comparing trees of the same size). The largest contribution to the distances will result from comparing the number of instances of the Label $1$ (tip) in two trees; this is necessarily larger than any other label copy number, and furthermore, a tree with more tips can have more cherries, pitchforks and any other subtree than a tree with fewer tips.

However, it is straightforward to construct metrics that compare tree shapes of different sizes with respect to their proportional frequencies of subtrees. We based the metric $d_2$ on vectors whose $i\text{th}$ components were the number of sub-trees of label $i$; we can divide these vectors by the number of tips $n_a$ in the tree: $\hat{v}_a = \tfrac{1}{n_a}v_a$, and include a component of $\hat{v}$ proportional to the number of tips ie $\hat{v}_a(0) = \epsilon n_a$. We then define a new metric, again based on the Euclidean norm,

$$d_{2}^{\prime }(T_{a},T_{b})=||{\hat{v}}_{a}-{\hat{v}}_{b}||$$

with $\epsilon > 0$. With small $\epsilon$, $d'_2$ will be small when the proportional frequencies of sub-trees are very similar (even if the trees are different sizes), but will only be 0 if the trees have identical vectors and the same number of tips. Furthermore, if there are particular labels $i$ that are of interest - for example those with relatively few tips, for a “tip-centric” tree comparison, weights $w$ can be chosen and applied to the vectors, such that the $i'th$ component of each vector is multiplied by the $i'th$ weight, $v^w_a(i) = w_i v_a(i)$, to emphasize some entries more than others :

$$d_{w}(T_{a},T_{b})=||v_{a}^{w}-v_{b}^{w}||.$$

The same weighting can of course be applied to $\hat{v}$ in $d'_2$.

The labeling scheme induces natural metrics on tree shapes, but does not capture the lengths of branches. These are biologically relevant in many examples, because they reflect the (inferred) amount of time or genetic distance between evolutionary events, although particularly for branches deep in the tree structure they may be difficult to infer accurately. Branch lengths or other features of trees—including the number of lineages through time, diversity measures, tip-tip distance profiles, imbalance measures and bootstrap values—can be incorporated into metrics based on our scheme. As our metric satisfies $d(T_1, T_2)=0 \iff T_1 = T_2$, any distance function of the form

(5) $$d(T_{1},T_{2})=w_{1}d_{i}(T_{1},T_{2})+w_{2}C(T_{1},T_{2}),$$

where $C(T_1,T_2)$ obeys the triangle inequality will be a metric (though not necessarily Euclidean), even if the features in the comparison $C$ do not uniquely define a tree. In Equation (5), $d$ is a metric if $C$ is a pseudo-metric.

**Figure S4:**
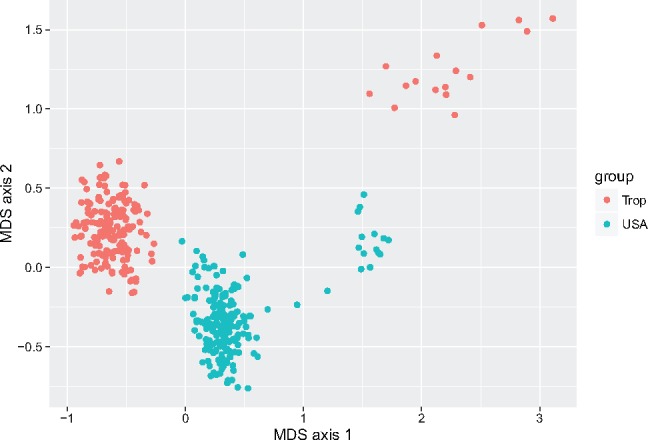
Multidimensional scaling plot derived from distances $\hat{d}$, containing a weighted combination of the shape distance and a length-based comparison of the ratio between the mean terminal branch length and the mean internal branch length in each tree.

We can create Euclidean metrics that combine lengths and other features with our shape comparisons. To do this, we describe trees $a$ and $b$ with vectors $V_a$ and $V_b$. The first $F$ components of $V$ capture $F$ comparable summaries or length-based statistics, and the remaining components count the label frequencies (as in $v$). Weights can be applied as above, component wise, to define $V^w_a$. In this way, we can create any number of Euclidean metrics

(6) $$\hat{d}(T_{1},T_{2})=||V_{a}^{w}-V_{b}^{w}||,$$

where $w$ reflects weightings across the label numbers and summary features. Summary features or comparisons could include spectral differences, Sackin or Colless imbalance, Kullback–Leibler divergence between lineages-through-time plots, maximum likelihood parameter estimates, mean bootstrap values, bootstraps corresponding to each shape label, or other features. Because $\hat{d}$ can only be equal to 0 if $T_1$ and $T_2$ are the same shape (because of the components of $V$ that include the shape label), $\hat{d}$ is a Euclidean metric. This extends the shape metric to incorporate branch lengths and to emphasize features of interest (believed to be informative of an underlying process), while retaining the advantages of a true distance metric. Supplementary Material Figure S4 available on Dryad illustrates this approach on the tropical and USA trees, showing an multi-dimensional scaling plot of $\hat{d}$, where the first component of $V$ is the ratio of the mean terminal branch lengths to the mean internal branch lengths in each tree. While the main shape separation between tropical and USA tree shapes is preserved, there is an informative length dimension illustrating the presence of outliers with high mean terminal branch length.

The labeling scheme maps tree shapes and natural numbers in a bijective way: each tree has a unique label (the label of its root node) and each natural number (positive integer) specifies a unique tree. In the Supplementary Material available on Dryad, we show how this can be used to map tree shapes bijectively to the integers and then to the rational numbers. Because addition and multiplication are defined on the integers we can use these maps to “add” and “multiply” trees, and to define a *convex* metric on tree shapes – a metric such that there is a tree shape directly in between any two distinct tree shapes. The convex metric allows us to compute averages of sets of trees by taking the averages of the corresponding rational numbers. Although this is the first convex metric defined on the space of tree shapes to our knowledge, its properties are not intuitive and it is left in the Supplementary Material available on Dryad for the interested reader.

## Discussion

We have developed metrics on unlabeled tree shapes, and used them to compare simulated and data-derived trees. The labeling scheme on which the metrics are based comprises a complete characterization of rooted tree shapes, and is not limited to bifurcating trees. Trees from processes known to produce different shapes are well separated in the metric that arises naturally from the scheme. This suggests applications in inferring evolutionary processes and to detecting tree shape bias (Huelsenbeck and Kirkpatrick 1996; Gascuel 2000; Stam 2002). The structure and simplicity of this comparison tool carry a number of advantages. Metrics have good resolution in comparing trees because the distance is only zero if tree shapes are the same. Empirical distributions of subtree shapes can easily be found and compared. And as we have shown, the approach can be extended to convex metrics on tree shapes, allowing averaging as well as algebraic operations (addition, multiplication) in tree space. However, this approach does not seem likely to give rise to analytically tractable distributions of tree–tree distances, and in some cases, may not offer more useful resolution than a well-chosen collection of summary statistics.

In particular, scalar measures of asymmetry perform well in distinguishing rooted binary trees. Here, imbalance measures perform slightly worse on the continuous-time BD models with $R0=2, 5$ but are different between the Yule, PDA, biased and Aldous’ random processes. (Matsen 2007) developed a method to define a broad range of tree statistics. Genetic algorithms uncovered tree statistics that can distinguish between the reconstructed trees in TreeBase (Sanderson et al. 1994) and trees from Aldous’ $\beta$-splitting model, whereas imbalance measures do not (Blum and François 2006). However, the search-and-optimize approach is vulnerable to over-fitting, as the space of tree statistics is large. It is also reasonable to believe that due to ongoing decreases in the cost of sequencing, studies will increasingly analyze large numbers of sequences and reconstructed trees will have many tips. Any single scalar measure will likely be insufficient to capture enough of the information in these large trees to perform inference.

Large trees present a problem for many approaches to inference including phylodynamic methods that rely on computationally intensive inference methods. In contrast, our scheme is better able to distinguish between groups of large trees than small ones (fewer than 100 tips). The tip-to-root traversal means that it is very efficient to construct the label set on very large trees (and the same traversal could, with little additional computation time, compute other properties that are naturally computed from tip to root, such as clade sizes, some imbalance measures and many of Matsen’s statistics (Matsen 2007)). However, due to the large number of tree shapes, the labels themselves become extremely large even for relatively small trees. Our implementation used MD5 hashing to solve this problem, but hashing removes the ability to reconstruct the tree from its label. Also, there are $2^{128}\approx 3\cdot10^{38}$ possible hashed strings, which while large is less than the number of possible tree shapes, even restricting to 500 tips. Alternative labeling schemes may partially alleviate this, for example by subtracting from the label the minimum label for $n$ tips, and only comparing trees of size $n$ or greater. A related approach was used by (Furnas 1984) in developing algorithms to sample trees.

The large size of the labels is also a challenge when they are mapped to the integers and rational numbers (see Supplementary Material available on Dryad) to define a tree algebra or a convex metric. Small changes in the label value can correspond to visible changes in the shapes, and small changes in a shape can correspond to large changes in the label. Because the bijective maps are sensitive to small perturbations, the implementation requires the full label, with no hashing compression. However, for trees with 500 tips, we encountered labels of about one million digits. Handling such large numbers with full accuracy required heavy and slow computation. The search for the average tree, using the convex map to the rational numbers presented in the Supplementary Material available on Dryad, was only possible for small trees, as the map requires the prime factorization of the label.

Our scheme captures only the shape of the trees; there does not appear to be a natural way to incorporate branch lengths other than appending statistics of branch lengths to the vectors describing the tree (as we have done, though this could be done for each label rather than in aggregate, with a cost for the size of the vector). There are several non-metric approaches to comparing unlabeled trees that do include lengths. In particular, Poon’s kernel method (Poon et al. 2013) compares subset trees that are shared by two input trees, after first “ladderizing” the trees (arranging internal nodes in a left-right order with branching events preferentially to one side). Using a kernel function, this approach can quantify similarity between trees. One challenge is that differences in overall scaling or units of the branch lengths can overwhelm structural differences. Lengths can of course be re-scaled (e.g., such that the height of both trees becomes 1), but results may be sensitive to outliers or to the height of the highest tip in the tree. Lengths could also be set to 1 to compare shapes only. Recently, Lewitus and Morlon (LM) (Lewitus and Morlon 2015) used the spectrum of a matrix of all the node-node distances in the tree to characterize trees; this is naturally invariant to any node and tip labels. They used the Kullback–Leibler divergence between smoothed spectra as a measure of distance. As it uses all node-node distances, this approach, requiring the spectrum of a non-sparse $2n-1 \times 2n-1$ matrix for a binary tree of $n$ tips, becomes infeasible for large trees.

One option is to add one or several terms to the distance function to incorporate more information, as outlined above. Combinations of our distances and other tree comparisons may turn out to be the most powerful approach to comparing unlabeled trees, allowing the user to choose the relative importance of scalar summaries, tree shape, spectra and so on while retaining the discriminating power of a metric. Ultimately, discriminating and informative tools for comparing trees will be essential for inferring the driving processes shaping evolutionary data.

## Supplementary Material

Supplementary Data

## Figures and Tables

**Figure S1:**
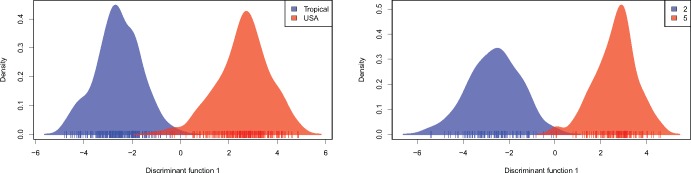
Discriminant analysis of principal components: One principal component separates the groups of trees in both cases. Left: tropical versus USA trees. Right: simulated birth–death trees.

**Figure S2:**
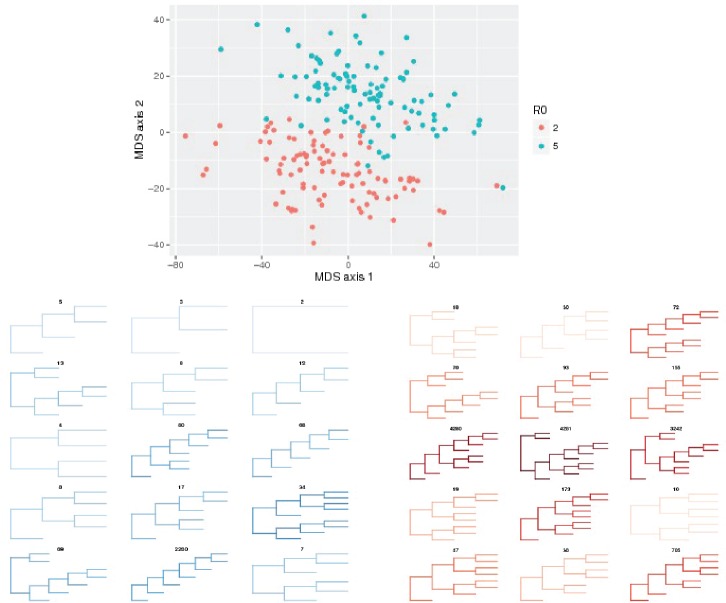
Multidimensional scaling plot comparing the trees from the simulated birth–death process. Lower: Tree shapes that distinguish the two groups as determined by discriminant analysis of principal components. Color represents groups in the lower panel, so the blue subtrees are more prevalent among the trees with $R_0=5$ and the red more prevalent in $R_0=2$. The trees’ integer labels correspond to the labeling scheme. Depth of color corresponds to Sackin imbalance, with darker shading corresponding to higher imbalance.

**Figure S3:**
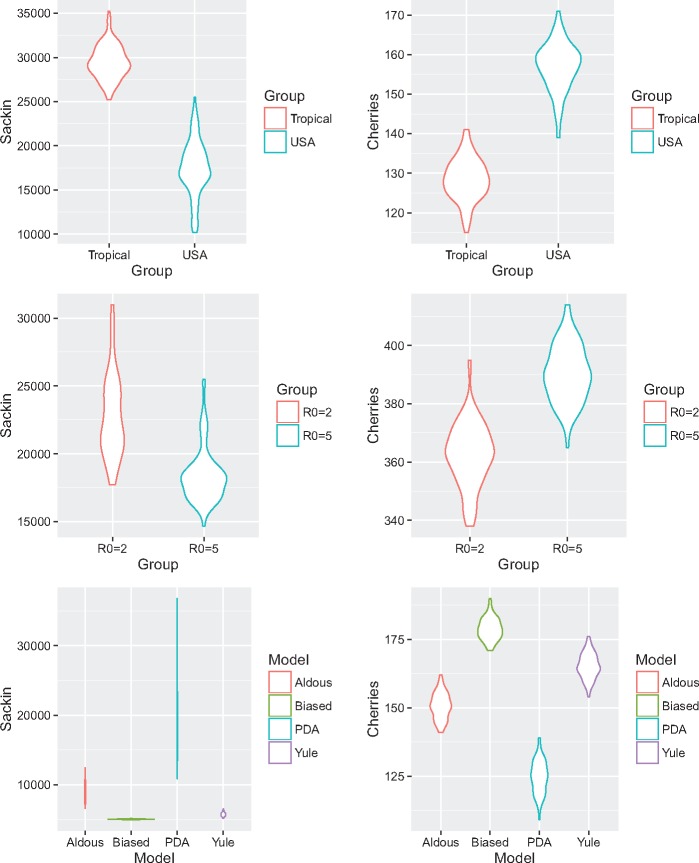
Two standard tree summary statistics, the Sackin imbalance and the number of cherry configurations, in the tropical and USA flu trees, the simulated birth–death trees with different values of the basic reproduction number $R_0$ and in the trees from different random models.

**Figure S5:**
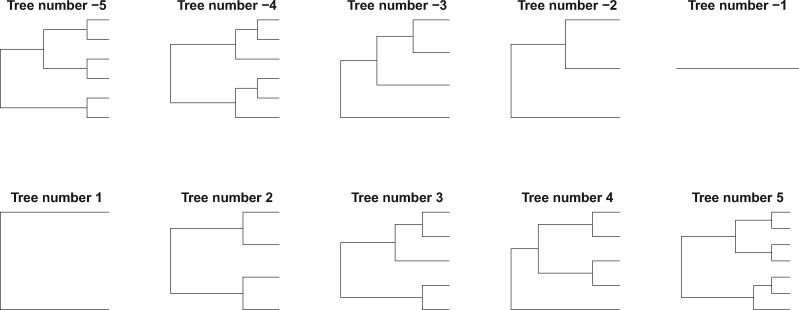
Some trees and their associated integers using the map $\psi_\mathbb{Z}$ of Example 1. The numbering goes from $-$5 to 5, with the exception of 0 which corresponds to the “empty tree”.

**Figure S6:**
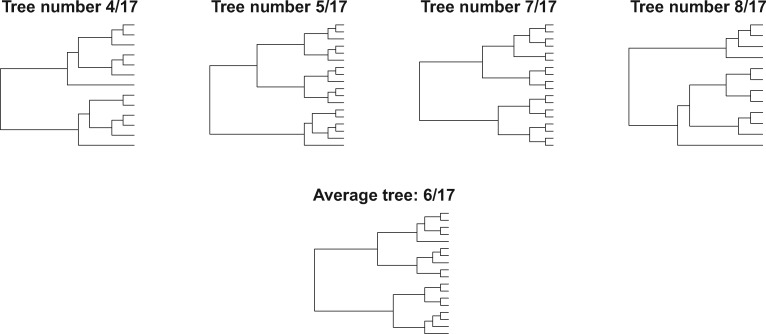
Trees associated with the rationals 4/17, 5/17, 7/17, 8/17, using the map in Example 2. Because the natural distance is convex in $\mathcal{Q}^+$, it is possible to find the “average” tree, which is the one mapped into 6/17. Moreover, trees mapped to 5/17, 6/17 and 7/17 are part of the direct path between the trees mapped to 4/17 and 8/17.

## Supplementary Material

Data available from the Dryad Digital Repository: http://dx.doi.org/10.5061/dryad.3r8v1

## Supplement for: A Metric on Phylogenetic Tree Shapes

### Supplementary Results

#### Trees Characteristic of Distinct Groups

The Euclidean nature of the $d_2$ metric allows techniques such as principal components analysis to be used to find low-dimensional representations of a set of trees. We have used discriminant analysis of principal components (Jombart et al. 2010) to determine which components (subtree shapes) best distinguish the tropical versus USA influenza trees, and the simulated birth–death trees with different birth-to-death ratios ($R_0$). In both cases, a single axis separates the groups of trees almost entirely; Fig. S1 illustrates this, showing the value of the first component.

Since there is only one principal component and it almost entirely separates the trees into two groups (this is the case both for the tropical versus USA and for the birth–death trees), it is straightforward to determine which subtrees make up this principal component and therefore best separate the groups. The loadings of the vector entries $v_i$, corresponding to trees $i$ (1 for a tip, 2 for a cherry and so on), reflect the importance of the $i'$th tree in distinguishing the groups. Figure S2 shows the subtrees that are more frequently present in the two groups of birth–death trees, in parallel with Fig. 3 in the main text (for the tropical versus USA trees).

Imbalance, or asymmetry, is the most widely discussed scalar measure of tree shape. Indeed, for rooted full binary trees, in the absence of branch length considerations, the most natural quantity to examine at each node is the difference between the numbers of descendants on the two sides (the key quantity in the Colless imbalance) and/or the path lengths from the tips to the root. Imbalance does a good job in separating the groups, but cannot in itself reveal which imbalanced subtrees are more highly represented in which groups.

We illustrate how the shape metric can be extended to include branch lengths and other features of the trees, even if those themselves are not metrics and do not uniquely define a tree. Let $V$ be a new vector, whose first $k$ components are various summary features or other properties. Here, let $V(1)$ be the ratio of the mean terminal branch length in a tree to the mean internal branch length. This captures how “star-like” a tree is, where star-like trees have long-terminal branches and short-internal ones. Let the remaining components of $V$ be the counts of the labels, as we have done throughout: number of 1s, 2s, 3s, and so on. Then the metric $\hat{d}$ is

(7) $$\begin{array}{cc} \hat{d}(T_{1},T_{2}) & =||w\cdot V^{a}-w\cdot V^{b}||\\ & =\sqrt{(w_{0}V_{1}^{a}-w_{2}V_{1}^{b}{)}^{2}+\sum_{i=1}^{L}w_{i}(v_{i}^{a}-v_{i}^{b}{)}^{2}}, \end{array}$$

where superscripts refer to which tree $a,b$ the entry is from, and subscripts $1$ and $i$ refer to the entry of the vector. $\hat{d}$ is Euclidean because it is the standard Euclidean distance between two vectors. The weights $w$ should be chosen to reflect the desired weighting and the natural scaling of different variables; the counts in $v$ are integers and the natural unit is “number of occurrences”; to compare these to branch lengths in substitutions per site requires a scaling choice. In Fig. S4, we use a weight of $w_0=1$ and $w_i= 0.00067$ for all $i$, to compensate for the fact that the mean branch length is much less than 1. Figure S4 illustrates that metric $\hat{d}$ retains the shape separation and additionally identifies similarity between outliers in the length statistic.

### Extension to multifurcations and sampled ancestors

A polytomy, or multifurcation, is an internal node with more than two children. In extending the scheme to handle polytomies, we also extend it to allow for internal nodes with only one child.

We first explicitly work out the case where the maximum size multifurcation is 4. Let $0$ be the empty tree. Nodes may have 0, 1, 2, 3, or 4 children, and we write a general tree as $(k,j,l,m)$, where $k$, $j$, $l,$ and $m$ are the labels of the four trees descending from the root. Some of these may be empty (0) as not every node is a $4$-fold polytomy. As in the binary case, we use the convention that $k \geq j \geq l \geq m$, and sort the length four strings lexicographically. Every possible tree $T$ with a maximum size multifurcation of four has a unique label $\phi_4(T)$ in this list. We seek to find an explicit expression for the label $\phi_4(T)$—the order in the list—for the tree $(k,j,l,m)$. We begin by fixing $k$ and finding how many such labels there are, going from $(k,0,0,0)$ up to $(k,k,k,k)$. Summing these over $m<k$ will give the explicit expression for the label.

The number of possible labels in the scheme with four characters, starting with $k$ and sorted lexicographically, is ${k+3 \choose k}$. To see this, note that each $(k,j,l,m)$ with $k \geq j \geq l \geq m$ can be thought of as a path on a lattice, starting on the left at height $k$ and descending to height $0$ after three horizontal steps. The path has a total length of $k+3$ steps, and of these, three must be steps to the right and $k$ must be downward. The number of such paths is the number of ways of placing three rightwards steps amongst $k+3$ steps, that is ${k+3 \choose k}$. Extending this, we obtain the label $\phi_4$ of the tree $(k+1, 0, 0,0)$, noting that $\phi_4(k,k,k,k)$ is the sum of the numbers of labels beginning with 1, 2, ... k. $\phi_4(k+1,0,0,0)= 1+\phi_4(k,k,k,k)$ (and we write 1 as ${3 \choose 3}$):

ϕ4(k+1,0,0,0)=∑x=0k(x+33).

Rewriting the sum and making use of the identity $\sum_{y=0}^{k+c} {y \choose c} = {k+c+1 \choose c+1}$, we have

ϕ4(k+1,0,0,0)=∑x=0k(x+33)=∑y=3k+3(y3)=∑y=0k+3(y3)=(k+44).

To obtain $\phi_4(k,j,l,m)$, we note that

$$\phi_{4}(k,j,l,m)=\phi_{4}(k,0,0,0)+\phi_{3}(j,0,0)+\phi_{2}(l,m)$$

(where $\phi_2$ is not precisely the same form as in the main text because here we allow for nodes with only one child). Following the same logic, this is

ϕ4(k,j,l,m)=(k+34)+(j+23)+(l+12)+m.

As in the binary case, the labels will grow unfeasibly large, but in principle this is a bijective map between trees whose maximum size polytomy is 4 and the nonnegative integers.

Naturally, there is nothing special about size four polytomies. If the maximum size is $c$, the scheme is

ϕc(xc,xc−1,xc−2,..,.x1)=∑i=1c(xi+i−1i).

### Mathematical extensions mapping trees to the integer and rational numbers

#### Addition and Multiplication of Tree Shapes Defined by the Mapping

Natural metrics associated with the labeling scheme are all based on the bijective map $\phi$ between the tree space $\mathbb{T}$ and the natural numbers $\mathbb{N}$. Composing $\phi$ with bijective maps between $\mathbb{N}$ and other countable sets like the integers ($\mathbb{Z}$), the positive rational numbers ($\mathbb{Q}^+$), or the rationals ($\mathbb{Q}$) opens up further possibilities because we can take advantage of the properties (addition, multiplication, distance, etc) of integer and rational numbers. If $\psi$ is a bijective map between $\mathbb{N}$ and one of these sets, then the composition $\psi\circ\phi$ is also bijective, and we can use it to define addition and multiplication operations on trees:

(8) $$\begin{array}{cc} & T_{1}⊕T_{2}=\phi^{-1}\psi^{-1}(\psi (\phi (T_{1}))+\psi (\phi (T_{2}))),\\ & T_{1}⊗T_{2}=\phi^{-1}\psi^{-1}(\psi (\phi (T_{1}))\cdot \psi (\phi (T_{2}))), \end{array}$$

where + and $\cdot$ are the usual addition and multiplication. Now the space of trees together with these definitions of addition and multiplication, $\bigr(\mathbb{T}, \oplus,\otimes \bigr)$, inherits all the algebraic properties of the set it is mapped into. For instance, $\bigr(\mathbb{T}, \oplus,\otimes\bigr)$ is a commutative ring if $\psi:\mathbb{N}\to\mathbb{Z}$. These constructions allow algebraic operations in the tree space $\mathbb{T}$. However the choice of the map $\psi$ determines whether these operations are “meaningful” or “helpful” for applications of branching trees in biology or other fields. It turns out that the selection of a meaningful map is challenging.

For example, we can use the labeling scheme to map tree shapes to the (positive and negative) integers.

We first extend $\phi$ with $\phi(0)=\emptyset$, that is the empty tree no tips. Consider the following well-known map between $\mathbb{N}$ and $\mathbb{Z}$:

$$\psi_{Z}:n\to \left\{ \begin{array}{cc} \frac{n}{2} & \text{ if }n\text{ is even}\\ -\frac{n+1}{2} & \text{ if }n\text{ is odd} \end{array}\right..$$

$\psi_\mathbb{Z}$ is clearly bijective: Each tree shape is mapped to a unique integer and each integer corresponds to a unique tree shape. A representation of 10 trees is provided in Fig. S5. To "add" or "multiply" trees, we can add or multiply their corresponding integers and then invert, as in Equation (2). This may seem intuitive for small trees; for example the sum of tree number 3 and tree number $-$1 gives tree number 2 which has one fewer tip than tree number 3. For larger trees, however, addition and multiplication operations are less intuitive and do not follow the numbers of tips. This map has the advantage of simplicity but results in a large distance between trees differing by one tip.

#### Mapping Tree Shapes to the Rational Numbers

We use the map to the integers, and a map to the rational numbers, to define a *convex* metric on tree shapes. Convexity is the property that there is a point directly in between two other points (so that equality holds in the triangle inequality). Define the following map from $\mathbb{N}$ to $\mathbb{Q}$: $\psi_{{\mathbb{Q}^+}}:n\to \prod_{i=1}^{\infty} p_i^{\psi_{\mathbb{Z}}(a_i)}\text{ if }n>0, \text{ or } 0 \text{ if } n=0$. Here, $p_i$ are all the prime numbers and $\prod_{i=1}^{\infty} p_i^{a_i}$ is the unique prime decomposition of $n+1$. $\psi_{\mathbb{Z}}$ is as defined above, mapping the positive integers to all integers. For example $\psi_{\mathbb{Q}^+}(11)=2^{\psi_\mathbb{Z}(2)}3^{\psi_\mathbb{Z}(1)}=2^{-1}3^1=2/3$. $\psi_{\mathbb{Q}^+}$ is injective, from the uniqueness of the prime factorization and the injectivity of $\psi_\mathbb{Z}$. Therefore it is also bijective, because $\mathbb{N}$ and $\mathbb{Q}^+$ have the same cardinality. Therefore $\psi_{\mathbb{Q}^+}\circ\phi$ maps tree shapes bijectively to the nonnegative rational numbers. In turn, $\mathbb{T}$ inherits all of the properties and structure of $\mathbb{Q}^+$. A distance metric $\mathcal{D}$ on $\mathbb{T}$ can be defined from the usual distance $|\cdot|$ of $\mathbb{Q}$:

$$D(T_{1},T_{2})=\left|\psi_{Q^{+}}(\phi (T_{1}))-\psi_{Q^{+}}(\phi (T_{2}))\right|.$$

Because the absolute value is a convex metric in $\mathbb{Q}$, this is a convex metric on unlabeled tree shapes. It can be used to find averages of a set of trees.

Figure S5 illustrates tree shapes together with their labels under the map $\psi_\mathbb{Z}$.

#### A Convex Metric on Tree Shapes

Mapping tree shapes to other sets of numbers can help us to capture the space of tree shapes in new ways. A particularly nice property of a metric space is convexity—if given two trees $T_1$ and $T_2$, there exists a tree $T_3$ lying directly between them, that is $d(T_1, T_3)+d(T_3,T_2)=d(T_1,T_2)$. Convex metrics are appealing because in a convex metric on tree shapes we can find the average tree shape for a set of trees, define a center of mass shape, and further develop statistics on the space of tree shapes.

We use the labeling scheme and a pairing of maps to construct a convex metric on tree shapes. To do this, we map tree shapes to the rational numbers, where the usual absolute value function is a convex metric (as there is always a rational number directly in between any two others). We use the prime decomposition, that is the unique product of prime factors of a number (e.g., $ 10 = 2 \cdot 5$). For a tree shape corresponding to integer $n$, we apply $\psi_\mathbb{Z}$ to the exponents of all the prime factors of $n+1$, and multiply the result (see Methods). For example $\psi_{\mathbb{Q}^+}(19)=2^{\psi_\mathbb{Z}(2)}5^{\psi_\mathbb{Z}(1)}=2^{1}5^{-1}=2/5$. We denote this map $\psi_\mathbb{Q}$; it takes each integer to a unique rational number, and vice versa (bijective).

Applying $\psi_{\mathbb{Q}^+}\circ\phi$ to tree shapes maps them bijectively to the nonnegative rational numbers. We can add or multiply trees’ corresponding rational numbers to perform operations in the space of tree shapes. In particular, we can use the usual absolute value distance function to define a convex metric space of tree shapes $\bigr(\mathbb{T},\mathcal{D}\bigr)$:

$$D(T_{1},T_{2})=\left|\psi_{Q^{+}}(\phi (T_{1}))-\psi_{Q^{+}}(\phi (T_{2}))\right|.$$

In this space, we can find the average tree of a group of trees, and a “direct path” between two trees. Given $n$ trees, the average tree is:

$$T_{m}=\phi^{-1}\circ \psi_{Q^{+}}^{-1}\left(\frac{\sum_{i=1}^{n}\psi_{Q^{+}}\circ \phi (T_{i})}{n}\right).$$

In other words, the average of a set of trees is the tree corresponding to the average of the trees’ rational numbers under the map we have defined. Figure S6 illustrates this operation.

There are infinitely many ways that we could map tree shapes to rational numbers and we have chosen one that is relatively easy to write down explicitly. Any of them would give rise to a convex metric on the set of tree shapes. It would be most desirable if the resulting metric had some intuitive features—for example, if the trees lying directly between trees $T_1$ and $T_2$ (with $n_1$ and $n_2$ tips) had an intermediate number of tips between $n_1$ and $n_2$ inclusively. The convex metric we have constructed does not have this property, and indeed, since the path between any two rationals traverses a countable infinity of other rationals, but there is a finite number of trees with between $n_1$ and $n_2$ tips, no such metric can exist. This convex metric also relies on the prime factorisation of the tree labels, which is a challenge if large labels are encountered.
