# Supplementary material for: A Metric on Phylogenetic Tree Shapes
Source: Syst Biol. 2017 May 2;67(1):113–26. doi: 10.1093/sysbio/syx046 (PMC5790134; doi:10.1093/sysbio/syx046)
Supplement: Supplementary Data [file syx046_supp.zip › allTrees.pdf]

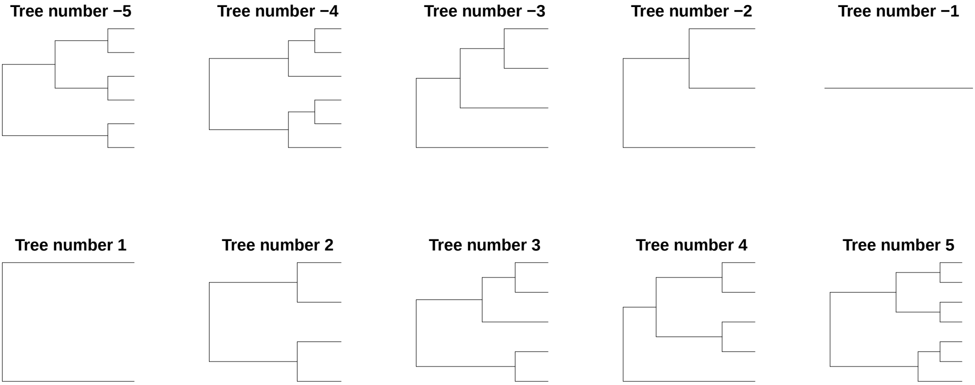
Tree number −5
Tree number −4
Tree number −3
Tree number −2
Tree number −1
Tree number 1
Tree number 2
Tree number 3
Tree number 4
Tree number 5
