# Supplementary figures and images for: A Metric on Phylogenetic Tree Shapes
Source: Syst Biol. 2017 May 2;67(1):113–26. doi: 10.1093/sysbio/syx046 (PMC5790134; doi:10.1093/sysbio/syx046)

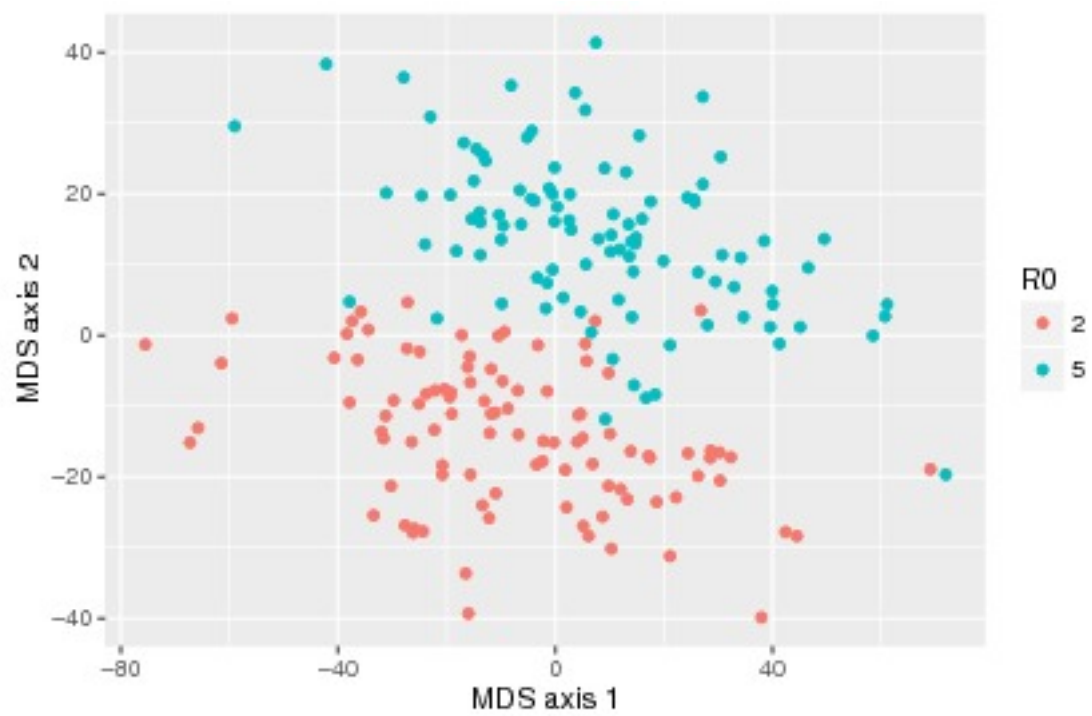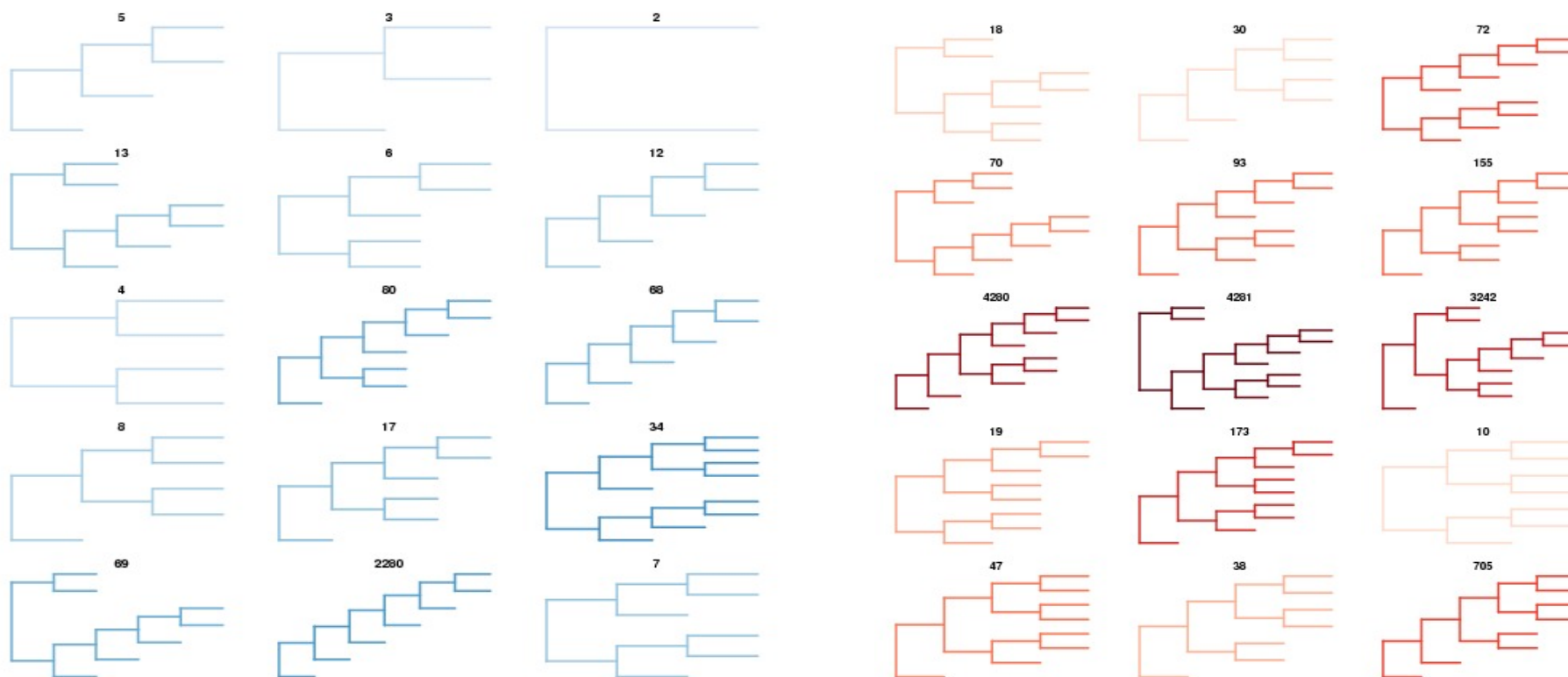

Supplement: Supplementary Data [file syx046_supp.zip › FigureS1.pdf]

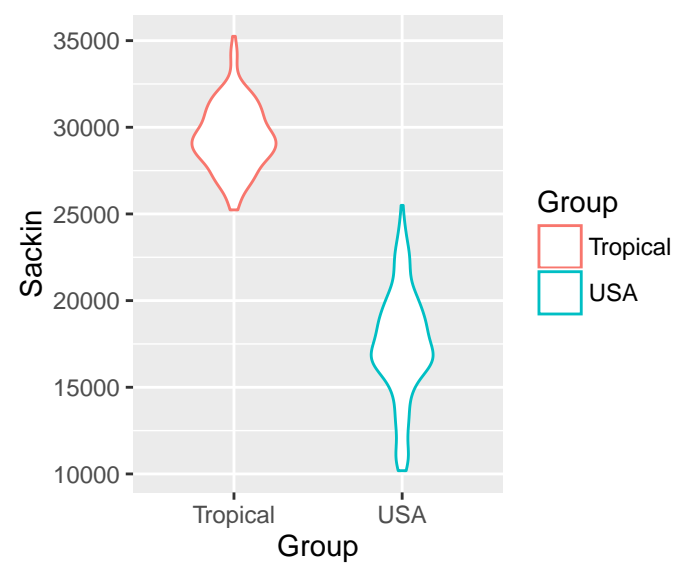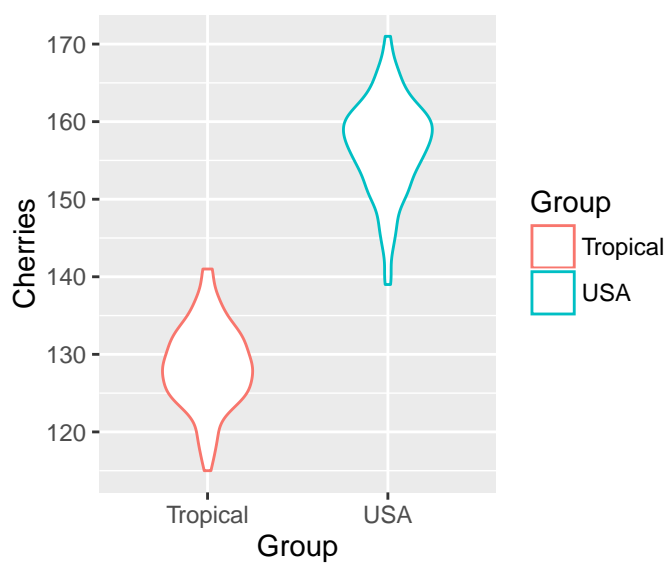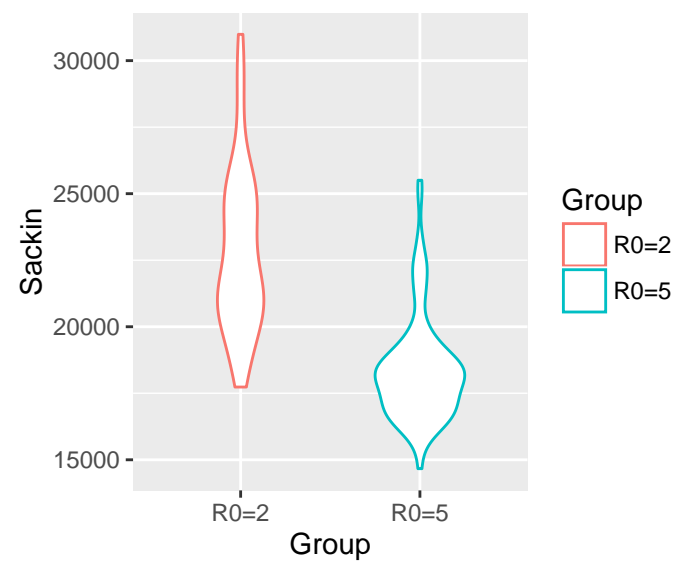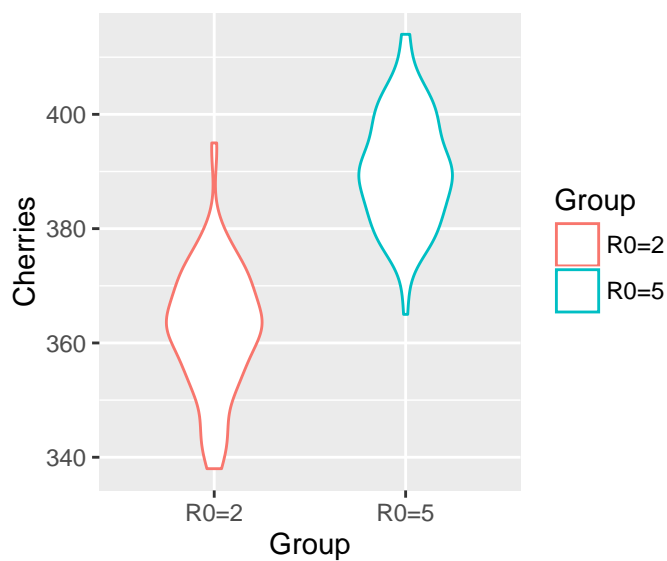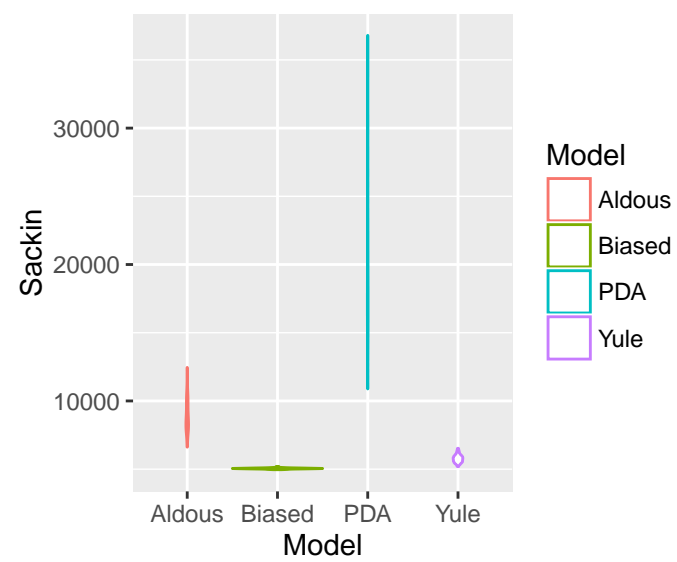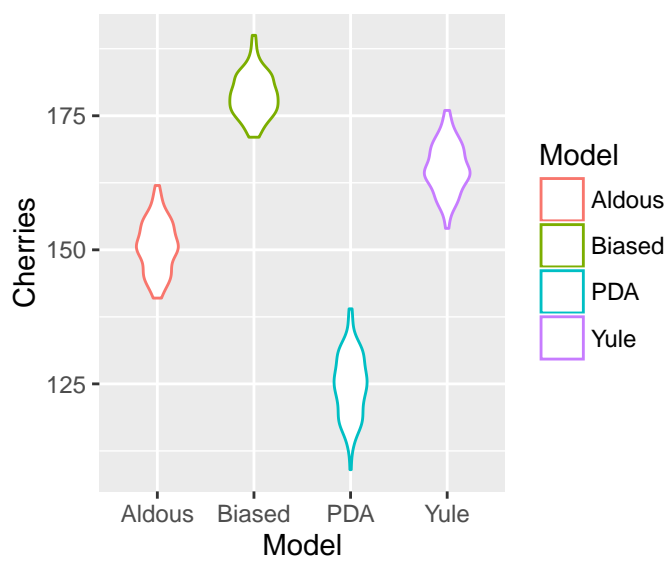

Supplement: Supplementary Data [file syx046_supp.zip › FigureS2r.pdf]

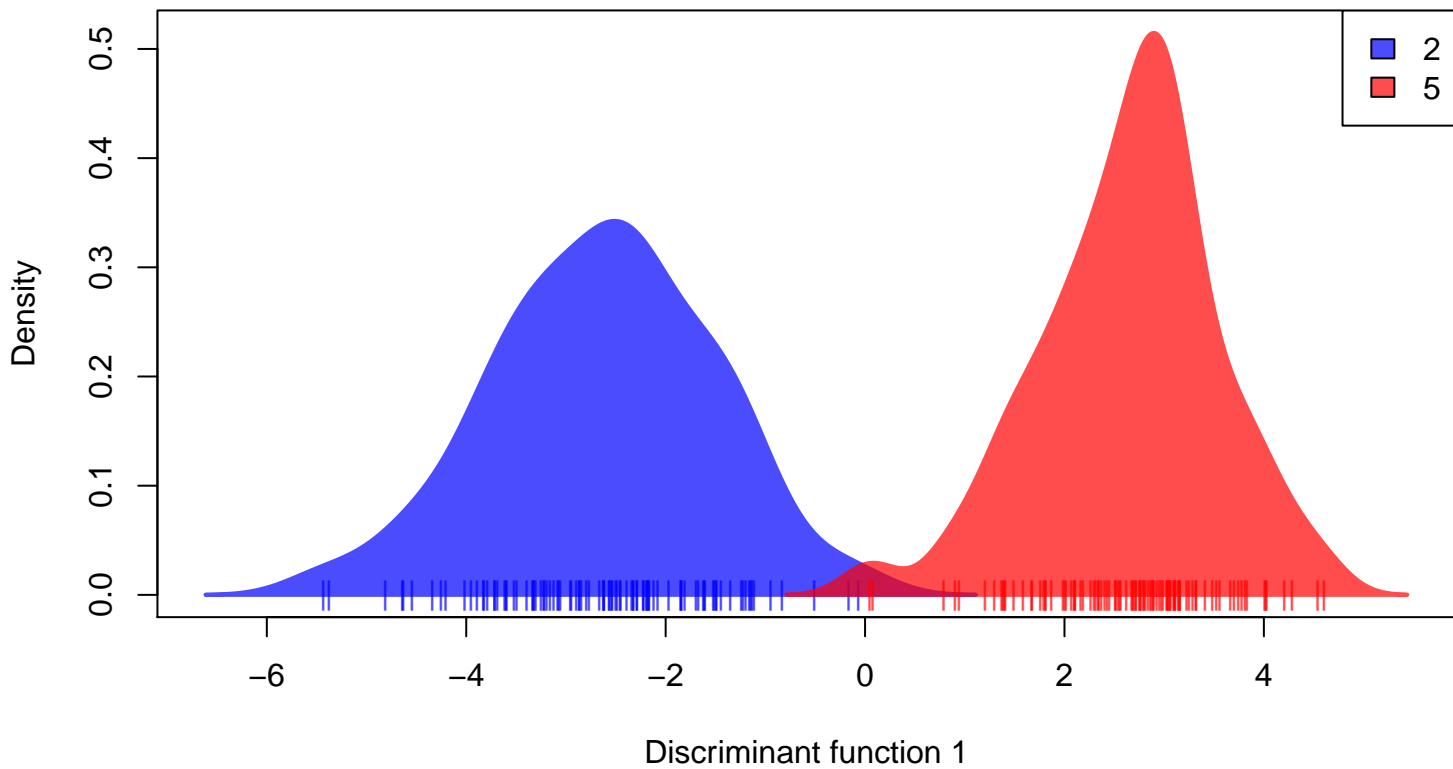

Supplement: Supplementary Data [file syx046_supp.zip › scatterDapcSimsRO.pdf]

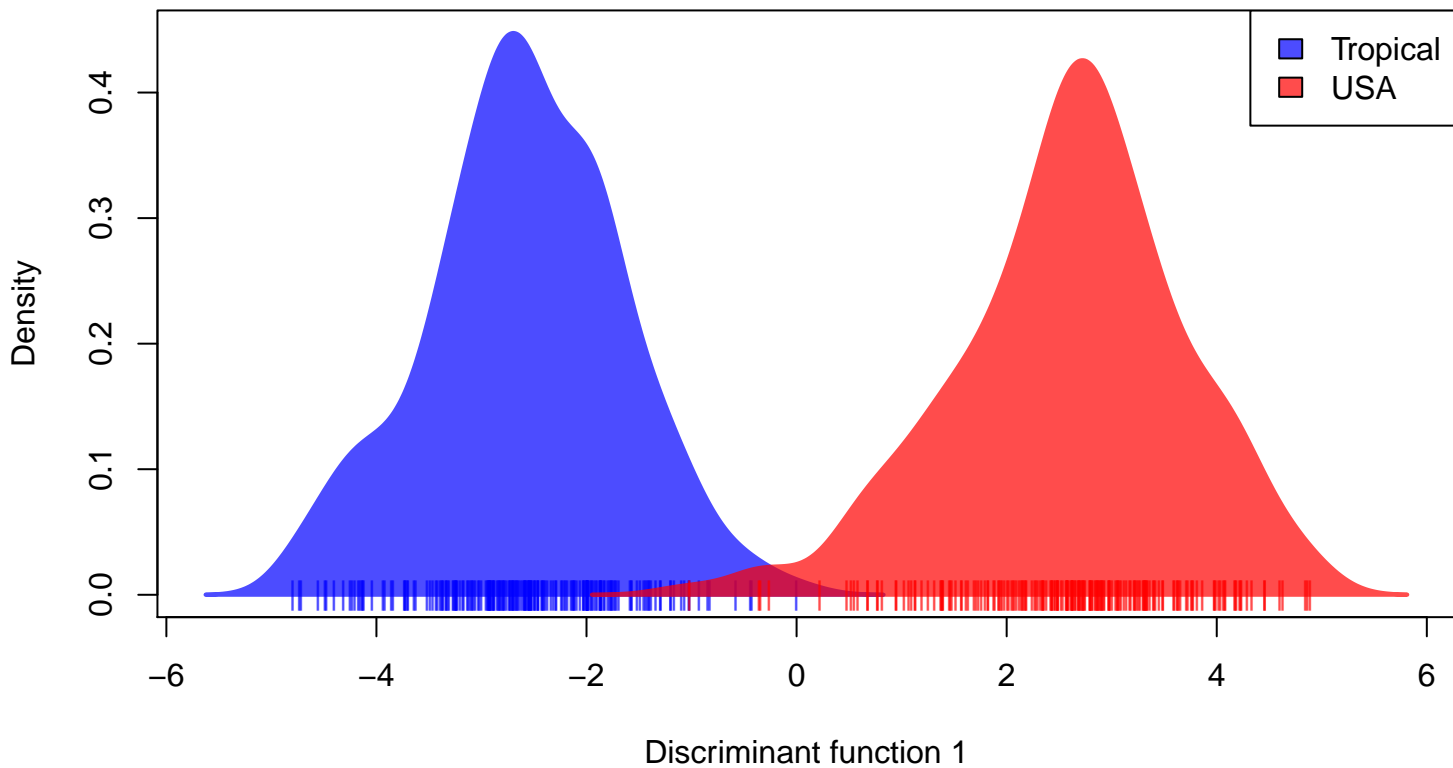

Supplement: Supplementary Data [file syx046_supp.zip › scatterDapcTropUSA.pdf]

**Tree number 4/17**

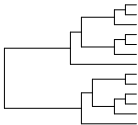

**Tree number 5/17**

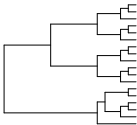

**Tree number 7/17**

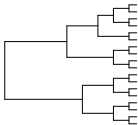

**Tree number 8/17**

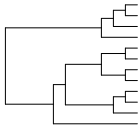

**Average tree: 6/17**

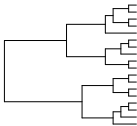

Supplement: Supplementary Data [file syx046_supp.zip › SFigure4-Qexample.pdf]

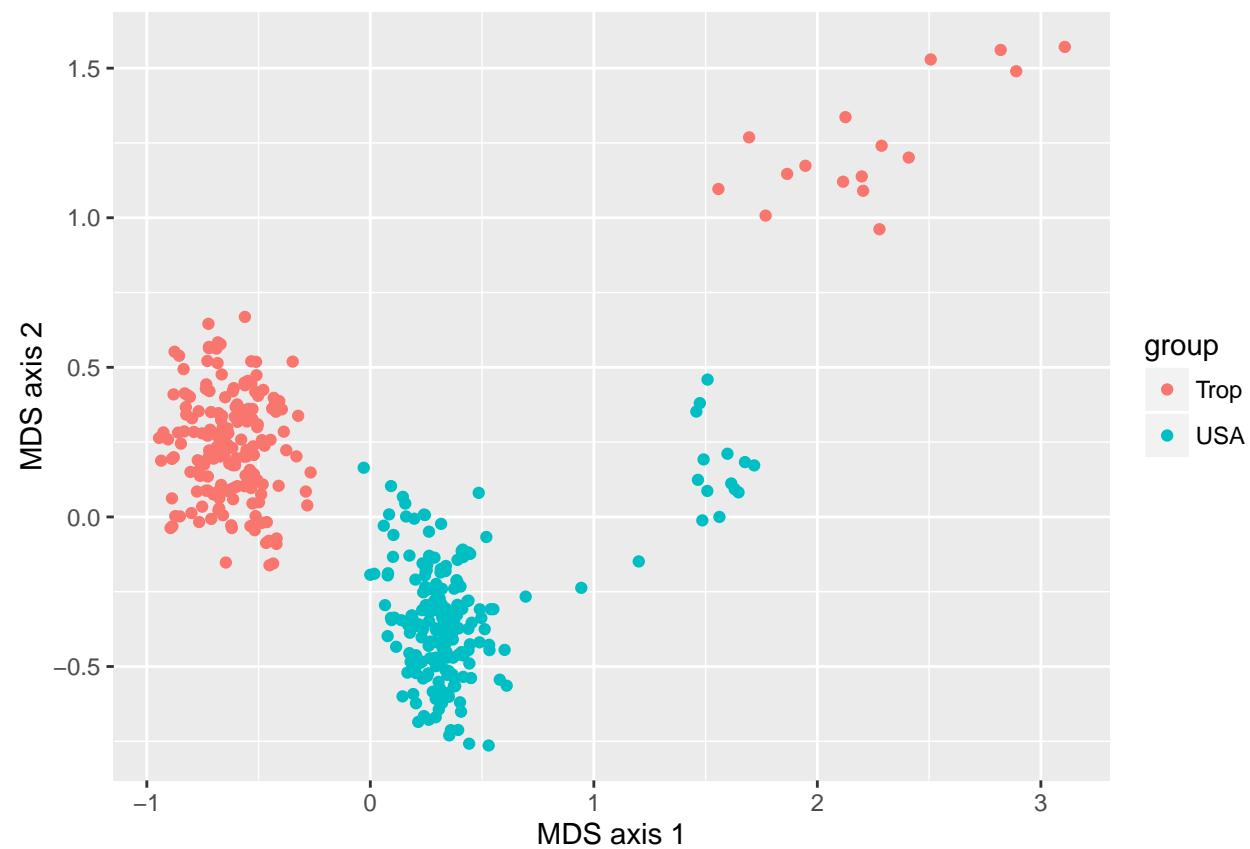

Supplement: Supplementary Data [file syx046_supp.zip › tropUsaMDSWithLength.pdf]
